# Third Dentition—Vicar. Mens.

**Published:** 1848-10

**Authors:** 


### "THIRD DENTITION—VICAR. MENS.

"Mary Ann, (a slave) supposed to be between 70 and 80, of active habits for her years, upwards of three years ago had a severe attack of pleurisy which left her very feeble, and last winter was affected with dysentery, attended with considerable pain. Her health however, became gradually established, and she appears to have acquired additional strength and vigor. The catamænia disappeared early in life. Two months ago, an incisor tooth made its appearance in the inferior maxilla, and a month afterwards having previously experienced pains about the back and loins, a sanguineous dark colored fluid was discharged from each nipple. There was no enlargement of the mammæ, nor was the discharge attended with pain, but she complained of weakness. It has now ceased."—S. Jour. Med. Sept., Wood's quarterly Retrospect, vol. 1, No. 2, page 94.

